# Role of Toll‐Like Receptors in Myeloid Neoplasms: Focuses on the Molecular Mechanisms and Clinical Impact on Myelodysplastic Syndromes, Acute Myeloid Leukemia, and Chronic Myeloid Leukemia

**DOI:** 10.1111/apm.70065

**Published:** 2025-09-09

**Authors:** Clarissa Brenda Alves Cavalcante, Alessandro Cavalcante Chaves, Vanessa Silva de Oliveira, Maria Amanda Silva de Araújo, Thayres Marinho Cunha e Silva, João Vitor Caetano Goes, Roberta Taiane Germano de Oliveira, Ronald Feitosa Pinheiro, Howard Lopes Ribeiro‐Junior

**Affiliations:** ^1^ Cancer Cytogenomic Laboratory, Center for Research and Drug Development (NPDM) Federal University of Ceara Fortaleza Ceara Brazil; ^2^ Post‐Graduate Program of Pathology Federal University of Ceara Fortaleza Ceara Brazil; ^3^ Post‐Graduate Program in Medical Science Federal University of Ceara Fortaleza Ceara Brazil; ^4^ Post‐Graduate Program of Translational Medicine Federal University of Ceara Fortaleza Ceara Brazil

**Keywords:** biomarkers, innate immune system, myeloid neoplasms, toll‐like receptors

## Abstract

Toll‐like receptors (TLRs) are essential components of the innate immune system, functioning as pattern recognition receptors (PRRs) to detect pathogen‐associated molecular patterns (PAMPs) and damage‐associated molecular patterns (DAMPs). In hematological malignancies, particularly myelodysplastic syndromes (MDS), acute myeloid leukemia (AML), and chronic myeloid leukemia (CML), TLRs influence inflammation, disease progression, and therapeutic response. This review highlights the prognostic relevance of TLR expression, the role of the MyD88 signaling pathway in clonal evolution, and the dual nature of TLR‐mediated immune responses, either promoting antitumor activity or contributing to leukemogenesis. Notably, TLR dysregulation in MDS and AML is associated with poor prognosis and genomic instability, whereas in CML, TLRs contribute to a protective microenvironment via NOD‐like and TNF‐α pathways. Therapeutic strategies targeting TLRs, including agonists and antagonists, show promise in enhancing antitumor responses, especially when combined with agents like purine nucleoside phosphorylase inhibitors. Furthermore, genetic variations in TLR pathways may influence individual susceptibility to infection and cancer progression, reinforcing the relevance of personalized medicine. Overall, this review underscores the need for continued research into TLR modulation as a foundation for innovative therapies in hematologic cancers.

## 1 Introduction

The innate immune system plays a fundamental role in the prevention, control, and elimination of infections caused by a variety of pathogens in the host, acting as the first line of defense against microorganisms. One of the key features of innate immunity is the presence of pattern recognition receptors (PRRs), which play a crucial role in this process. PRRs are responsible for identifying pathogen‐associated molecular patterns (PAMPs), specific molecules from each pathogen, damage‐associated molecular patterns (DAMPs), released when sterile injury occurs, and intracellular molecules are released into the extracellular environment, and xenobiotic‐associated molecular patterns (XAMPs), which are foreign chemical compounds that can also trigger immune responses [1, 2]. Unlike adaptive immunity, innate immunity acts immediately, is not specific to the pathogen, and does not generate immunological memory. Detection of PAMPs and DAMPs by PRRs leads to an increase in the transcription of genes involved in inflammatory responses, activating receptors of innate immunity, such as Toll‐like receptors (TLRs) [3, 4].

Toll‐like receptors are a class of transmembrane type I proteins related to innate immunity [5, 6]. These receptors possess antimicrobial and pro‐inflammatory activity and are present on the plasma membrane or within endosomal structures of immune cells such as dendritic cells, hematopoietic stem cells (HSPCs), endothelial cells, B lymphocytes, macrophages, neutrophils, and other cellular groups [3, 7]. There are two subgroups of TLRs, classified according to their cellular location and respective ligands. The cell surface‐expressed TLRs include TLR1, TLR2, TLR4, TLR5, TLR6, TLR10, which often form a gene cluster (TLR6‐TLR1‐TLR10) and primarily recognize components of the microbial membrane, such as lipids, lipoproteins, and proteins [8], and TLR11, which primarily recognize components of the microbial membrane, such as lipids, lipoproteins, and proteins. The other subgroup comprises TLRs 3, 7, 8, and 9, which are exclusively expressed in cellular vesicles such as the endoplasmic reticulum (ER), endosomes, lysosomes, and endolysosomes, and these receptors recognize microbial nucleic acids [9].

Signaling mediated by TLRs plays a critical role in hematopoiesis. Their dysfunction is related to various hematological malignancies and the emergence of myeloid disorders, contributing to the development of cancers, especially myeloid neoplasms. This signaling, generated mainly by the TLR‐MyD88 interaction in the antigen‐presenting cell, is extremely important for inducing the immune response, which interaction results in the production of a variety of pro‐inflammatory cytokines and chemokines, which can favor tumorigenesis by stimulating cell proliferation and migration, as well as creating a favorable microenvironment for tumor cells [10]. However, there is a scarcity of studies that detail the role of TLRs, particularly in relation to therapeutic approaches for MDS, AML, and CML.

This narrative review study aims to explore and synthesize, through the most recent studies, the complex and multifaceted role of TLRs in the pathogenesis of onco‐hematological diseases, highlighting how these elements of the innate immune system influence both the progression and prognosis of various types of cancer, with a particular focus on the need for more in‐depth investigation into their relationship with onco‐hematological diseases.

## 2 Toll‐Like Receptors and PRRs: How Does the Immune Response Play Its Role?

**FIGURE 1:**
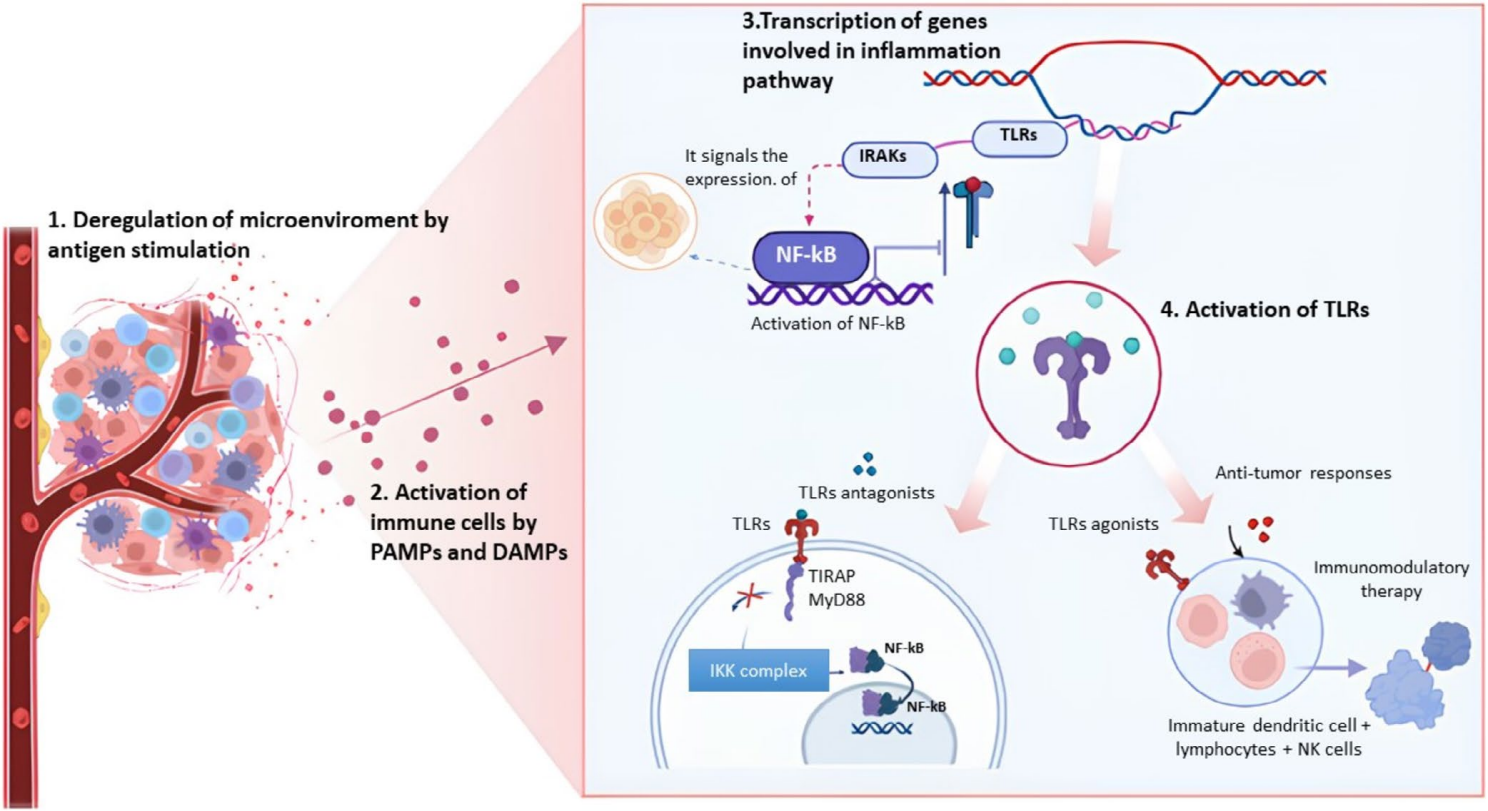
In the tumor microenvironment (TME), Toll‐like receptors (TLRs) play a dual role in cancer pathogenesis. (1) DAMPs released from necrotic tumor cells and PAMPs from the tumor‐associated microbiota. (2) Activate immune cells. These trigger signaling cascades, such as the MyD88‐dependent pathway, leading to the activation of transcription factors like NF‐κB. (3) In neoplastic conditions, the sustained activation of NF‐κB is a key mechanistic driver of tumorigenesis, as it promotes the transcription of genes involved in cell proliferation, survival (anti‐apoptosis), angiogenesis, and metastasis. (4) This highlights the therapeutic potential of modulating TLR activity. (5) TLR antagonists can block this chronic pro‐tumorigenic inflammation, while TLR agonists are used in immunomodulatory therapy to activate dendritic cells (DCs) and natural killer (NK) cells, promoting a robust antitumor cytotoxic T‐cell response.

Innate immunity represents the first line of defense in humans (Figure 1). This response is characterized by the activation of PRRs, which identify PAMPs or DAMPs [11]. This triggers inflammatory processes through various immune pathways, including the activation of the interferon pathway, TLRs, and the formation of inflammasomes. While TLRs are versatile sensors on the cell surface and in endosomes, other PRR families have specialized roles; for example, RIG‐I‐like receptors (RLRs) are cytosolic sensors crucial for detecting viral RNA and initiating a potent interferon response, while certain NOD‐like receptors (NLRs) form the inflammasomes mentioned, which are critical for processing pro‐inflammatory cytokines like IL‐1β [8, 12].

TLRs are found in various cells and play an essential role in antimicrobial and pro‐inflammatory responses, recognizing PAMPs and DAMPs to induce inflammation and phagocytosis. When a PAMP binds to a PRR, various activation pathways in the cell are triggered, leading to changes in protein synthesis, altering gene expression, and producing molecules with antimicrobial and pro‐inflammatory functions to eradicate the microorganism. Some molecules can recognize these patterns and are freely soluble in plasma, such as pentraxins, a protein involved in the process of acute immune responses, as well as collections‐mannose‐binding lectin, ficolin, and the complement system, which are capable of opsonization [13].

TLRs, part of a pattern recognition receptor family, can engage with endogenous DAMPs and trigger the innate immune response. Furthermore, TLRs are critical signaling molecules that orchestrate immune responses, playing an important role in host defense against pathogens and maintaining immune equilibrium [14]. This critical role in deciding the fate of an infection is exemplified in viral diseases such as COVID‐19, where the timely activation of TLRs (e.g., TLR3 and TLR7) is essential for a protective antiviral response, while the dysregulated hyperactivation of others (e.g., TLR2 and TLR4) can trigger a pathological “cytokine storm,” leading to severe immunopathology and acute respiratory distress syndrome (ARDS) [8, 12].

Furthermore, the clinical outcome of infections varies among individuals, a phenomenon partly explained by genetic polymorphisms in TLR genes. Single nucleotide polymorphisms (SNPs) can alter receptor function, leading to hypo‐ or hyper‐responsive inflammatory states that influence susceptibility or resistance to disease [15]. For instance, the D299G SNP in TLR4 is known to modulate host responses and has been associated with the severity of various infectious and inflammatory conditions. The TLR7 gene has been linked to severe COVID‐19 in males, highlighting how an individual's genetic background can impair critical immune pathways and affect disease outcome [15].

Thus, to bridge the discussion of the innate immune role of TLRs with their therapeutic potential, it is essential to consider how these receptors not only initiate protective responses but also influence disease progression when dysregulated. The dual nature of TLR activation, as both a defender against pathogens and a contributor to pathological inflammation, sets the stage for exploring innovative therapeutic strategies. Understanding how to harness or modulate these pathways provides a foundation for the development of targeted therapies aimed at restoring immune balance and combating disease progression, as discussed in the following section.

## 3 Therapies Based on TLRs: How Can We Modulate the Immune Response?

The involvement of TLRs in the pathogenesis of human cancers has drawn attention to the development of therapeutic approaches focusing on the regulation of TLRs in cancer cells. This perspective has built two different views on the application of TLR modulators: one focusing on TLR suppression (TLR antagonists) and the other on the immunomodulatory role of TLRs in inducing antitumor responses (TLR agonists). So far, over 1000 compounds have been developed in clinical trials to evaluate their anticancer properties along with their safety [16].

Before discussing therapeutic modulators, it is necessary to understand the diverse and often dual roles different TLRs play in the pathogenesis of various cancers. The article by Mukherjee et al. [17] provides a comparative view, highlighting that TLR expression is frequently dysregulated in tumors. For instance, in brain cancers like glioma, activation of TLR2, TLR4, and TLR9 promotes tumor growth and metastasis by modulating signaling pathways such as NF‐κB and MAPK. In lung cancer, TLR4 activation can induce proliferation, while TLR7 and TLR8 signaling contributes to chemoresistance. Similarly, in breast cancer, TLR2 is linked to chemoresistance and metastasis, while TLR4 overexpression promotes cell proliferation and invasion. A common immunopathogenic mechanism across many of these cancers is the activation of the MyD88/NF‐κB axis, which drives the secretion of pro‐inflammatory cytokines (e.g., IL‐6, TNF‐α), promotes angiogenesis, and upregulates anti‐apoptotic genes, thereby creating a tumor‐supportive microenvironment [17].

Currently, there are two categories of developed TLR antagonists, known as direct and indirect TLR inhibitors. Direct TLR antagonists were primarily developed as structural analogs of agonists that prevent the agonistic action of TLR ligands by binding them to the receptor and blocking downstream inflammatory/autoimmune cascades [18]. Indirect TLR inhibitors, on the other hand, are a group of anticancer agents that can override the signaling associated with TLR4. As illustrated in Table 2, the best example of these agents is TAK‐242, also known as Resatorvid, a small molecule that binds to the TIR domain and prevents TLR4 from interacting by blocking LPS‐induced signaling [18]. This signaling blockage limits the formation of an immune response, reducing the individual's inflammatory status, hindering DNA damage, and clonal proliferation.

TLR agonists are essentially considered potent immunomodulators with the ability to activate innate immunity and initiate long‐lasting adaptive immunity by stimulating cytotoxic lymphocytes, natural killer cells (NK cells), and inducing the maturation of dendritic cells (DCs). Given these unique characteristics, TLR agonists can be recruited for cancer treatment as monotherapy or in combination modalities [18]. Although all evidence suggests that agonists have effective therapeutic value in cancer treatment, it should be noted that not all TLR agonists and not all TLR signaling pathways lead to clinically relevant antitumor activity. In fact, the successful clinical development of TLR agonists requires careful selection of agents because the activation of some TLRs in tumor cells has been shown to increase tumor growth and metastasis [11].

In hematologic malignancies, the aberrant expression and signaling of TLRs are also well‐documented and present therapeutic opportunities. Studies have reported dysregulated expression of TLRs in lymphomas, leukemias, and multiple myeloma. For example, genetic variants in the TLR1/6/10 gene cluster and in TLR9 have been associated with an increased risk for non‐Hodgkin's lymphoma. In chronic lymphocytic leukemia (CLL), low TLR2 expression is linked to a poor prognosis, while in mantle cell lymphoma (MCL), TLR4 signaling can trigger tumor growth. This dysregulation often supports cancer cell survival and proliferation through canonical pathways like NF‐κB, which promotes the autocrine secretion of survival factors such as IL‐6, particularly in multiple myeloma. These findings underscore the potential for TLR‐targeted therapies in various hematological cancers [17].

Abegunde et al. [34] investigate how the presence of TNF‐α in an inflammatory environment favors the expansion of TET2 mutant HSPCs. The results show that these cells have a clonal advantage, with greater resistance to apoptosis and a shift in the generation of myeloid lineages to the detriment of lymphoid ones, driven by inflammatory cytokines such as IL‐6 [34]. These findings have significant clinical implications, suggesting that normalizing the inflammatory microenvironment may be a promising strategy to control clonal hematopoiesis associated with aging and hematological diseases, highlighting the importance of modulating the immune environment in the management of these conditions.

The central role of TLRs in immune response and tumor pathogenesis underscores their significance as targets for therapeutic development. Therapeutic strategies involving TLRs include antagonists that inhibit receptor activation and agonists that enhance immune responses, aiming to modulate inflammatory pathways and promote antitumor effects. Additionally, an innovative therapeutic approach explores the development of vaccines against tumor‐associated microbial antigens, such as the Fap2 protein from *Fusobacterium nucleatum* in colorectal cancer, where the vaccine is designed to potently activate host TLRs and generate a robust antitumor immune response [35]. However, the dual role of TLRs, as either tumor suppressors or promoters, necessitates careful selection and design of modulatory agents. This complexity highlights the importance of precision in targeting TLR pathways to optimize therapeutic outcomes in hematological malignancies while minimizing unintended effects such as enhanced tumor growth or metastasis.

## 4 TLRs In Hematological Tumors—Same Gene, Different Pathways Modulations

To date, autoimmune and chronic inflammatory diseases, such as rheumatoid arthritis, systemic lupus erythematosus (SLE), and psoriasis, have been thoroughly associated with the development of hematologic neoplasms. Here, we explore the multifaceted roles of TLRs in hematological malignancies, with TLR9 serving as a prime example of the diverse impact of TLRs on the occurrence of hematological tumors. By analyzing its varied roles across different diseases, we provide insights into its dual impact on tumor progression and prognosis.

For example, Rizzello et al. [36] evaluated the relationship between the inflammatory process generated by SLE and the appearance of diffuse large B cell lymphoma. Their studies showed that the osteopontin linked to the pathogenesis of Lupus can be a decisive factor in the malignant transformation of the B cell, when this protein is defectively expressed in lymphoid tissue, also when there is polymorphic variation of its gene in light of constant inflammatory stimuli originated by TLR9‐MyD88 signaling [36]. In fact, numerous reports have pointed out that a high TLR9 expression is linked to tumor expansion and metastasis, establishing its identification as a new prognostic biomarker in the context of hematological cancer, although its role in prognosis stratification is not still fully established [37].

In this instance, highlighting another modulation by the TLR9, this time in a study about angioimmunoblastic T‐cell lymphoma carried out by Qian and collaborators revealed that individuals exhibiting elevated PD‐1 expression experienced a poorer overall survival (OS), and those with high expression of both PD‐1 and PD‐L1 demonstrated an even more unfavorable prognosis. Notably, a significant proportion of these patients exhibited heightened TLR9 expression, underscoring an intrinsic connection between TLR9 expression and PD‐L1 expression in the disease, supporting previous proposals by Chen and collaborators. This co‐occurrence suggests a correlation with a worse prognosis, shown in Table 1 [43]. These findings collectively highlight the possible importance of TLR9 in hematological malignancies, providing valuable information for future prognostic considerations and therapeutic strategies, and illustrating how the same gene can generate different modulations in a disease in different ways.

**TABLE 1:** Summary of molecular data from studies related to TLRs and treatments.

TLR	Disease	Findings	References
TLR2	AML	Prevention of TLR2 or Akt/mTOR signaling attenuated MDSC differentiation, improving immune response	[38]
TLR3	MDS	Heightened TLR3 expression was linked directly and indirectly to adverse prognostic markers in MDS	[39]
TLR3	AML	The triggering of TLR3 in CD8α + dendritic cells activates transcription factors that result in the transcription of INF‐β, increasing the immune response	[40]
TLR4	MDS	The expression of TLR4, induced by the S100A9 protein, reduces the process of cellular senescence, reducing the rate of apoptosis of neoplastic cells	[41]
TLR4	CML	Reduction of TLR4 limited the action of the NF‐κB signaling axis and the generation of reactive oxygen species (ROS)	[21]
TLR5	AML	TLR5 combined with αCD40 improved dendritic cell (DC) maturation	[42]
TLR9	Angioimmunoblastic T‐cell lymphoma (AITL)	High expression of TLR9 or PD‐L1 indicated a low survival rate for patients with AITL, being unfavorable prognostic factors for the disease	[43]
TLR9	MDS	Ox‐mtDNA and TLR9 may provide a possible therapeutic target for patients with MDS, with possible blockade of TLR9 signaling	[44]
TLR2 and TLR4	AML	High TLR2 expression reduced OS and DFS compared to those with lower expression levels. Similarly, high TLR4 expression correlated with worse OS and DFS	[45]
TLR2 and TLR4	MDS	The expression levels of TLR2 and TLR4 were significantly increased in MDS compared to some studied non‐MDS malignancies (*p* < 0.05)	[46]
TLR3, TLR4 and TLR9	AML	Patients with favorable or intermediate‐risk AML were more likely to carry a certain allele in the TLR9 gene. As well as related TLR3 and TLR4 alleles at the level of metastasis	[38]

Another important TLR, expressed on myeloid cells and neoplastic cells, the TLR2 mediates the recognition of harmful molecules, leading to inflammation and activation of the MyD88/NF‐κB signaling pathway, which promotes tumor cell proliferation and survival. Furthermore, NF‐κB activation enhances the expression of anti‐apoptotic proteins, promoting tumor cell resilience against therapeutic interventions. Thus, TLR2's role extends beyond pathogen recognition as it actively shapes the tumor microenvironment by fostering inflammation [47].

However, this same activation of TLR2 can have adverse consequences, because although the initial stimulation of the immune response can increase antitumor immunity, it can also contribute to the formation of an immunosuppressive microenvironment, since TLR2 influences regulatory immune cells that can inhibit the immune response against the tumor. This duality of functions makes TLR2 a “two‐faced factor” in which its activation can both facilitate defense against cancer and promote tumor progression [47].

## 5 TLRs In Myelodysplastic Neoplasms: Innate Immunity Dysregulation May Lead to Disease Progression

**FIGURE 2:**
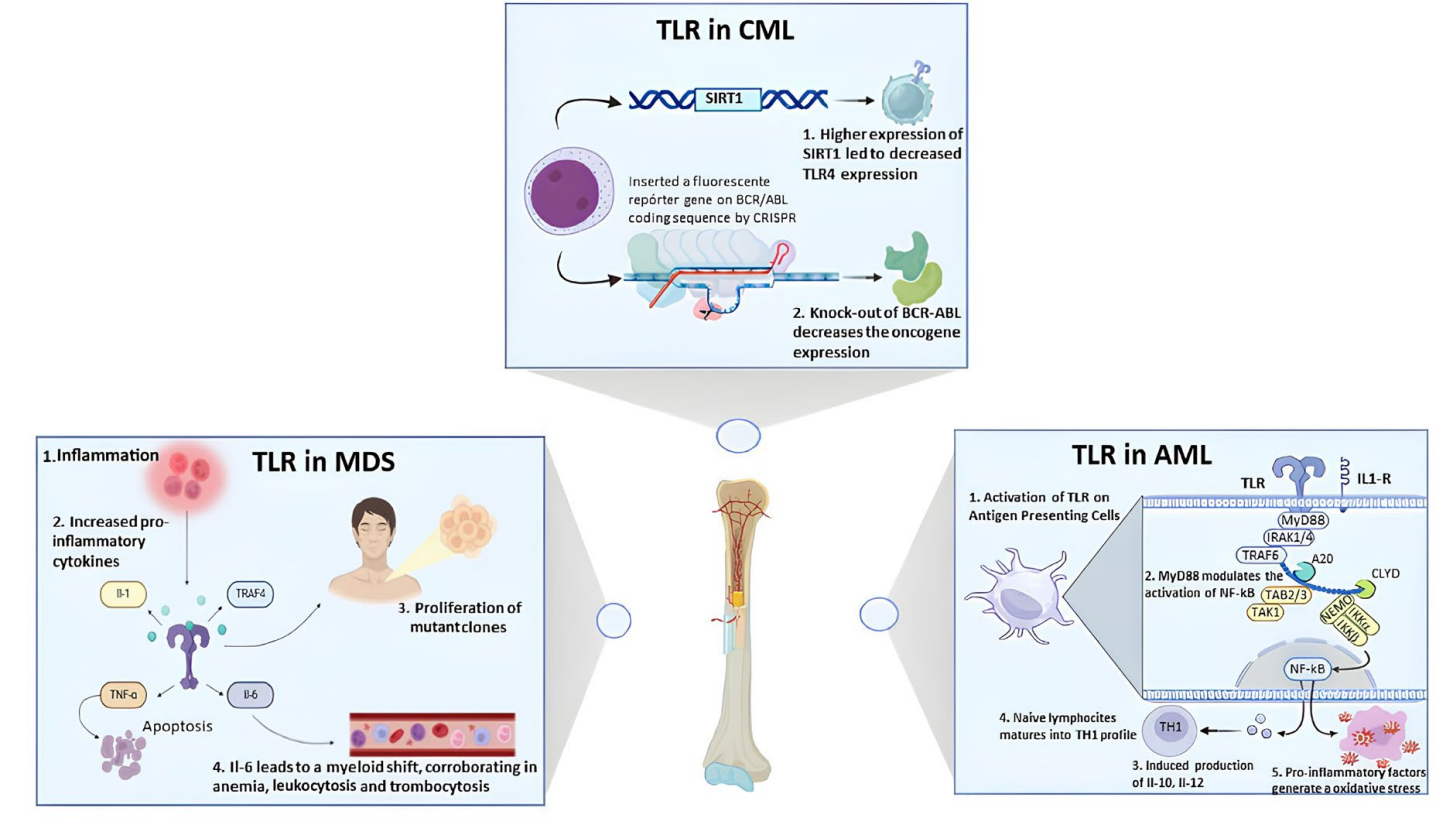
Mechanistic roles of TLR signaling in the pathogenesis of myelodysplastic and myeloproliferative neoplasms. In myelodysplastic syndromes (MDS), chronic TLR activation (e.g., via the TRAF6 pathway) in hematopoietic stem and progenitor cells creates an inflammatory bone marrow microenvironment. The resulting pro‐inflammatory cytokines, such as TNF‐α and IL‐6, mechanistically drive the disease by creating a selective pressure that favors the proliferation of mutant clones while inducing apoptosis in healthy hematopoietic cells, leading to ineffective hematopoiesis and characteristic cytopenias (anemia, leukopenia, and an increase in thrombocytosis). In acute myeloid leukemia (AML), TLR signaling through the MyD88‐NF‐κB pathway establishes a pro‐leukemic feedback loop. The activation of this pathway in leukemic blasts and antigen‐presenting cells induces the production of cytokines (e.g., IL‐1β, IL‐6) that promote the survival and proliferation of AML cells while creating an oxidative stress environment that suppresses normal hematopoiesis. In chronic myeloid leukemia (CML), the inflammatory TME, partly sustained by TLR signaling, supports leukemic cell survival. A key regulatory mechanism involves the deacetylase SIRT1; its overexpression has been shown to transcriptionally suppress TLR4 expression, providing negative feedback that can dampen this pro‐leukemic inflammation.

Myelodysplastic Neoplasm (MDS) is a heterogeneous group of clonal diseases characterized by disturbances in the bone marrow microenvironment, where high levels of pro‐inflammatory cytokines can also be found [48]. In fact, inflammatory diseases often proceed to MDS and preleukemic states, given that they are correlated with dysregulation of innate immunity signaling pathways [48]. Possibly caused by environmental stress, infections, and inflammatory processes, as shown in Figure 2, it directly contributes to the aging of hematopoietic cells, inducing manifestations of poor bone marrow in patients with MDS. Consequently, this leads to the appearance of classic clinical features of the disease, such as ineffective hematopoiesis in one or more myeloid lineages, with a risk of progression to acute myeloid leukemia (AML) [49, 50].

As illustrated in Table 2, MDS cells display dysregulated immune signaling due to altered NF‐kB pathways, which, within the mutated cellular context, are driven by non‐canonical activation. This alteration has significant implications for the pathogenesis and evolution of MDS, since the exacerbated activation of these immune pathways can contribute to a favorable microenvironment for tumor proliferation, especially in favor of the proliferation of myeloid clones through the activation of NF‐kB, thus contributing to the ineffective hematopoiesis commonly observed in MDS. Furthermore, Li et al. [25] uncovered that dendritic cells situated in perivascular clusters adjacent to the bone marrow express heightened levels of TLR1 and TLR2. Moreover, elevation of systemic TLR1/2 agonist levels induces the expansion and mobilization of hematopoietic stem and progenitor cells (HSPCs), concomitant with a shift from lymphopoiesis to myelopoiesis. This process also entails the conversion of osteoblasts into bone‐lining cells, regulated by IL‐1β in the microenvironment.

**TABLE 2:** Summary of data from several studies on TLR modulation, with emphasis on therapeutic approaches.

Type of study	Disease	Population	Main objective	Results	Comments
Preclinical Study	AML	2 human donors and 45 mice	Enhance the effector function of CAR‐T cells anti‐CD123 via activation of MyD88 and CD40	I. Tumor burden monitoring: Tumor burden tracked by serial bioluminescence imaging [19] CD123.ENG.iMC T cells showed significant antitumor activity in both donors [19] CD123.ENG.iM T cells controlled the tumor in only one out of two donors [19] CD123.ENG.iC T cells had no survival advantage compared to CD123.ENG T cells [19] CD19.ENG.iMC T cells demonstrated no antitumor activity, highlighting CID's antigen‐dependent efficacy II. Survival analysis CD123.ENG.iMC T cells provided a significant survival advantage for both donors [19] CD123.ENG.iM T cells showed a survival advantage for one donor [19] CD123.ENG.iC T cells did not exhibit a survival advantage compared to CD123.ENG T cells [19] III. Long‐Term Observations: Mice without tumors maintained stable weight for long‐term survivors [19] Some mice in the CD123.ENG.iM and CD123.ENG.iMC groups had to be euthanized due to non‐tumor‐related issues [19]	It was demonstrated the effectiveness of CD123.ENG.iMC T cells in antitumor activity, emphasizing the importance of antigen specificity and the role of CID in enhancing therapeutic outcomes
Preclinical Study	Clonal Hematopoiesis	4 human cells and murine cell model system	Analyzing an inflammatory state associated with TET2 inactivation and/or unhealthy aging may favor hematopoietic stem and progenitor cells (HSPC) with a TET2 mutation in the presence and absence of TNF‐alpha	In the presence of TNF‐alpha, BM TET2 −/− cells showed a growth advantage, while wt colonies decreased with increasing TNF‐alpha. TET2 −/− counts were maintained at TNF‐alpha concentration, but in its presence it has a replication advantage. TET2 −/−, TET2+/− and haploinsufficient cells showed superior replication capacity compared to controls under prolonged TNF‐alpha stress. HSPCs deficient in TET2 have a greater capacity to replicate and generate new colonies than wt cells [20]	Inflammatory cytokines are associated with the inflammatory state of myelodysplastic neoplasia, and it could be investigated whether the mutation in TET2 arises from inflammatory stresses. By conferring advantages, resistance to apoptosis in MDS becomes an avenue of investigation for therapeutic targets
Preclinical Study	CML	Cell culture (CML k562 cells)	Use lipopolysaccharide (LPS) to trigger inflammation in CML k562 cells and the role of SIRT1 as well as that of the Toll‐like receptor 4 (TLR4)–nuclear factor κB (NF‐κB)–reactive oxygen species (ROS) signaling axis in inflammation was investigated	The stimulation by lipopolysaccharide (LPS) mainly increased the expression of sirtuin 1 (SIRT 1), which led to a decrease in the expression of TLR4 (reduction in the production of pro‐inflammatory cytokines). This reduction in TLR4 ended up limiting the action of the nuclear factor κ B (NF‐κB) signaling axis and the generation of reactive oxygen species (ROS) [21]	The negative association between SIRT 1 and TLR4 decreases the action of inflammatory responses through the NF‐κB pathway, which may help regulate inflammation in neoplastic conditions
Clinical Trial	CML	Human	Identify a gene signature predictive for TFR using whole transcriptome expression analyses	Patients in the recurrence group showed increased expression of TLR1, TLR6 and TLR8 receptors, so that NOD‐like and TNFα signaling were increased (protective CML microenvironment) [22]	The increase in TLR1, TLR6, TLR8 together with the increase in NOD‐like and TNFα, contribute to the activation of the inflammatory gene that promotes the patient's relapse
Preclinical Study	CML	Cell culture (CML k562 cells)	Evaluate the ability of a CRISPR‐Trap system to direct by homologous recombination (HDR) a gene targeting strategy for specifically trapping BCR/ABL oncogene expression	Annulled the expression of the oncogene by inserting a fluorescent reporter gene into the BCR/ABL coding sequence in order to select the edited hematopoietic cells of CML and decrease of over 80% in BCR/ABL expression levels [23]	The CRISPR‐Trap strategy allows specific action on the BCR/ABL oncogene, providing promising results in the treatment of CML patients, especially those who do not have a satisfactory response to tyrosine kinase inhibitors
Clinical Trial	MDS	Human	To compare the safety and efficacy of hypomethylating agents, decitabine and azacitidine, in patients with low‐risk myelodysplastic syndromes (MDS) and associated myeloproliferative neoplasms	The analysis revealed that the presence of chromosome 5 deletion was associated with a more favorable clinical profile, with an improved therapeutic response compared to other MDS subgroups [24]	Although there are adverse effects, azacitidine becomes a good alternative for the treatment of MDS, as it is associated with an increased survival rate and a more favorable clinical profile
Experimental study	MDS	Mice	To investigate how TLR activation in bone marrow dendritic cells induces IL‐1β expression and how this cytokine regulates HSC function	TLR2 agonists induce the expression of IL‐1β in dendritic cells in the BM, in addition to having an impact on the functions of HSCs, activating them and promoting expansion and mobilization. Manipulation of the TLR2 pathway may improve HSC function in MDS [25]	These findings highlight the importance of interactions between the innate immune system and hematopoietic stem cells, opening new perspectives for therapeutic interventions in hematologic diseases
Experiment al study	AML	Mice	To develop and evaluate the efficacy of VacT2BP as a vehicle for targeted delivery of daunorubicin, taking advantage of the ability of TLR2‐binding peptides to induce a pro‐inflammatory response that potentiates antitumor activity	VacT2BP have been shown to be effective in delivering daunorubicin, increasing the concentration of the drug in target cells and improving its antitumor efficacy, in which TLR2 activation facilitated drug delivery, and in the animal model used there was a significant decrease in tumor growth [26]	The findings with VacT2BP have potential as a novel therapeutic strategy to improve the efficacy and reduce the adverse effects of daunorubicin treatment in hematologic cancers
Preclinical study	AML	Human cell	To investigate the correlation between the Toll‐like receptor 4 (TLR4) signaling pathway and the pathophysiological characteristics of patients with acute myeloid leukemia (AML)	Gene expression analysis by qRT‐PCR revealed that genes associated with the TLR4 pathway were significantly more expressed in patients with worse prognosis and drug resistance, in addition to the fact that treatment with TAK‐242 inhibited the proliferation of AML cell lines [20]	The TLR4 pathway is related to the pathophysiological characteristics of AML and its inhibition with TAK‐242 may be a promising strategy for the treatment of AML
Experimental Study	MDS	Mice	To determine how activation of the TLR2/6 signaling pathway influences the expansion of hematopoietic stem cells expressing the NUP98‐HOXD13 fusion gene, characterizing the role of this signaling in the pathogenesis of MDS	The findings suggest that interventions targeting the modulation of TLR2/6 signaling may be considered in the development of novel therapeutic approaches for MDS patients, addressing malignancy‐associated cellular expansion [27]	These results highlight the importance of TLR2/6 signaling in hematopoietic stem cell biology in MDS and open possibilities for new treatments targeting this pathway
Experimental study	AML	Mice	To evaluate the effects of combination therapy with BEZ235 and R848 on the modulation of the antitumor immune response and cell proliferation in a murine model of AML	Combination therapy resulted in a significant increase in the frequency of CD4+ T cells and M1 macrophages, which are associated with antitumor responses, while there was a decrease in pro‐tumor immune cells, such as MDSCs and M2 macrophages [28]	The results indicate that the combination of BEZ235 and R848 may be a powerful therapeutic option for patients with AML, promoting an effective immune response and combating disease progression
Experimental study	AML	Mice	Explore how the activation of pDCs can induce the death of AML cells through fratricide mechanisms, seeking to understand the interaction between the innate immune system and tumor cells, in addition to evaluating the therapeutic potential of this interaction	The activation of pDCs may be a viable therapeutic approach to increase the efficacy of AML treatment, by promoting the selective death of tumor cells and potentially improving clinical outcomes in patients. In addition, the combination of pDC activation with chemotherapeutic agents showed a synergistic effect, increasing treatment efficacy and suggesting that pDC [29]	These results suggest that modulation of pDCs may be a promising strategy to improve therapeutic responses in AML
Experimental study	MDS	Genetically modified zebrafish	To determine how TLR7 activation, mediated by the agonist imiquimod, influences hematopoiesis in zebrafish deficient in Rps14, a gene associated with del(5q) myelodysplastic syndrome (MDS)	The findings suggest that modulation of the TLR7 pathway may offer a novel therapeutic approach to treat anemia associated with Rps14 deficiency and other MDS‐related conditions, highlighting the potential of imiquimod as a therapeutic agent [30]	These results highlight the importance of TLR7 activation in promoting hematopoiesis and modulating the inflammatory response in pathological conditions associated with MDS
Clinical Trial	AML and ALL	Human	To evaluate the immune response caused by the TLR9 agonist GNKG168 in children with acute leukemia and minimal residual disease, in addition to determining the safety of the treatment and the resulting immune activation profile	The study reported that GNKG168 was well tolerated by children, with limited and manageable side effects, indicating that the treatment may be safe for this population, and that some data suggest that GNKG168‐induced immune activation may be associated with a reduction in leukemic cell burden [22]	This study highlights the importance of immunotherapy in the treatment of acute leukemia, especially in children, where the search for less toxic and more effective options is critical. The success of GNKG168 could represent a significant advance in the management of this challenging condition
Experimental study	MDS	Human cel and murine model	To determine whether modulation of the innate immune system through inhibition of HMGB1 could reduce cell viability in MDS, promoting cell death in malignant cells and altering immune responses associated with the disease	Treatment with the inhibitor sivelestat resulted in a decrease in MDS cell expansion, increased cell death, and preservation of healthy hematopoietic cells. After 17 weeks, MDS‐L cell engraftment in treated mice was significantly reduced compared with the control group. Sivelestat promoted apoptosis in MDS cells, and the combination of this drug with chemotherapeutic agents such as azacitidine or decitabine resulted in an additive increase in apoptotic cell death [31]	These results may represent a new approach to promote cell death in MDS and alter the immune responses associated with the disease, paving the way for new therapeutic strategies. In addition, the potential of HMGB1 as a therapeutic target in myelodysplastic syndrome offers a new perspective for the management of this complex condition
Experimental study	MDS	Mice	To investigate the effects of low‐grade inflammation on HSPCs in a murine model of del(5q) MDS, focusing on dysregulation of TLR signaling and activation of the TRAF6 pathway	Administration of IRAK1/4 or UBE2N inhibitors restored red blood cell counts and slightly improved neutrophil counts in the treated mice, suggesting that increased TRAF6 signaling is associated with the cytopenias observed [32]	The results found by the researchers may indicate that modulating TRAF6 signaling could be a promising therapeutic strategy for treating hematological complications in patients with del(5q) MDS
Experimental study	MDS	Mice	To understand how deregulated innate immune signaling with a TRAF6‐UBE2N inhibitor contributes to the transformation of an MDS‐like disease into AML, especially in the context of mutations in the RUNX1 gene	Inhibition of TRAF6‐dependent signaling using a UBE2N inhibitor can suppress leukemic HSPCs, underscoring the therapeutic potential of targeting innate immune pathways in AML, further highlighting the critical role of TLR4 signaling in maintaining the inflammatory microenvironment that promotes leukemic progression [33]	The study suggests that modulating innate immune signaling, especially by inhibiting the activation of TLRs, could be a promising therapeutic approach for treating MDS and preventing its progression to AML

Hence, the heightened TLR1/2 signaling implicated in the pathogenesis of MDS is noteworthy, given its upregulation in patients' dendritic cells and monocytes [25]. Mediated by A20 messenger and reliant upon TLR‐TRAF6 interaction, A20's deubiquitinating (DUB) activity selectively removes K63‐linked ubiquitin chains from TRAF6, thus inhibiting NF‐kB canonical pathway activation through TLRs. Moreover, A20 inhibits canonical NF‐kB activation induced by TAK1. Consequently, dysregulated non‐canonical NF‐kB activation, prompted by heightened immune stimuli, compromises the function of key genes that orchestrate the immune response, frequently found mutated and overexpressed in such scenarios [51]. This suggests the ability of dendritic cells to respond to stimuli that can activate the signaling pathways that lead to the production of inflammatory cytokines, and that this activation can be a factor in the progression of malignant diseases, where tumor cells can exploit these pathways to evade immune detection.

Individuals diagnosed with MDS commonly exhibit increased innate immune signaling, typified by elevated TLR2 and TLR4 expression levels observed in CD34+ HSPCs from 15 patients who experienced hypomethylating agent (HMA) therapy failure, as investigated by Paracatu et al. [52]. This failure resulted in impaired colony formation, ineffective hematopoiesis, and the emergence of dysplastic traits, hallmarks of the disease [31, 53]. Utilizing time‐of‐flight cytometry (cyTOF) to assess TLR expression in low‐ and intermediate‐risk MDS patients' bone marrow revealed augmented TLR expression in crucial innate immunity cells, including monocytes, granulocytes, B and T cells, and NK cells, in comparison to healthy counterparts. Conversely, high‐risk patients exhibited diminished TLR expression, indicative of compromised mechanisms governing dysplastic cell proliferation and reflected by altered receptor expression's impact on cytokine production and release, leading to exacerbated MDS progression in these instances [52].

Indeed, hematopoiesis exhibits high sensitivity to microenvironmental changes, displaying adaptability to various factors, including inflammation. Here, a critical player in inflammation, TLR2, operates in conjunction with either TLR1 or TLR6. Monlish et al. [27] demonstrated a correlation, in Table 2, between elevated TLR6 levels and high‐risk MDS, while TLR6 loss is associated with improved survival rates. Additionally, TLR2/6 stimulation exacerbates disease progression and amplifies the expansion of malignant HSPCs [27]. It could be suggested that this alteration in the microenvironment leads to uncontrolled proliferation of these cells, contributing to an increase in the malignant cell load and hindering the therapeutic response.

In addition, Khalilian et al. [46], searching for potential biomarkers in MDS patients, noticed increased levels of TLR2 and TLR4 mRNA in the bone marrow of MDS patients in comparison to healthy controls, possibly relating to an increased expression of these genes in individuals with the disease, demonstrated in Table 1. In addition, the authors also identified higher levels of TLR2 mRNA in the MDS group when compared to other hematological neoplasms, such as CLL and multiple myeloma, despite no difference being noted in comparison to AML. This suggests that the amplification of these mRNAs may be related to increased expression of these genes, whose roles have already been demonstrated in the pathogenesis of MDS, indicating their importance as possible biomarkers for diagnosis and prognosis of the disease, as well as a potential relationship within the evolution of MDS to AML [46]. This stimulation of TLR2/6 may imply disease progression, as there is an amplification of the expansion of malignant HSPCs, but it is also associated with better survival rates, which suggests that tighter control of inflammatory signaling may benefit patients, highlighting the importance of understanding the mechanisms underlying the activation of TLRs to develop more effective therapeutic strategies.

Furthermore, Li et al. [25] uncovered that dendritic cells situated in perivascular clusters adjacent to the bone marrow express heightened levels of TLR1 and TLR2. Moreover, elevation of systemic TLR1/2 agonist levels induces the expansion and mobilization of hematopoietic stem and progenitor cells (HSPCs), concomitant with a shift from lymphopoiesis to myelopoiesis. This process also entails the conversion of osteoblasts into bone‐lining cells, regulated by IL‐1β in the microenvironment. Hence, the heightened TLR1/2 signaling implicated in the pathogenesis of myelodysplastic syndrome (MDS) is noteworthy, given its upregulation in patients' dendritic cells and monocytes [25].

Following the association of these inflammatory markers with disease prognosis, Oliveira et al. [39] demonstrated in Table 1, in a cohort of 79 MDS‐diagnosed patients, a correlation between dyserythropoiesis and elevated TLR3 expression compared to patients lacking dyserythropoiesis, implicating TLR3 deregulation in erythroid lineage dysplasias in MDS. Additionally, heightened TLR3 expression was linked directly and indirectly to adverse prognostic markers in MDS, suggesting its pivotal role in bone marrow failure and ensuing severe anemic conditions and exacerbating transfusion dependence, underscoring its significance in bone marrow disorders [39].

Secondarily, dysregulation of the medullary microenvironment, evidenced by heightened inflammatory status in MDS, compromises mesenchymal stromal cells (MSCs), pivotal for osteogenesis and hematopoietic precursor interaction, thereby fostering tumor proliferation [54]. In this context, MACROH2A1 (MH2A1.1), a histone variant regulating epigenetic and MSC inflammation interaction in MDS, exhibited that its accumulation in MDS‐MSCs prompted pro‐tumoral TLR4 activation, ensuing in pro‐inflammatory mediator production and regulation that could impair the hematopoietic system's ability to support diseased cells [54]. One of the TLR4 activating genes is the S100A9 protein, which is responsible for inducing cell senescence in MSCs and regulating the tumor microenvironment. A study by Shi et al. [41] showed that this protein is highly expressed in low‐risk MDS, and the expression of TLR4, also induced by S100A4, showed an attenuation of the cellular senescence process, revealing a probable reduction in the apoptosis rate of neoplastic cells, exhibited in Table 1, whose expression of TLR4, induced by the S100A9 protein, reduces the process of cellular senescence, decreasing the rate of apoptosis of neoplastic cells, also contributing to the progression of the disease [41].

In the study by Varney et al. [32], the authors investigate the role of TLR4 signaling in the context of TIFAB deletion, a gene located within the commonly deleted region (CDR) of chromosome 5q, which is frequently altered in MDS and AML. The loss of TIFAB leads to dysregulation of hematopoiesis, primarily through its impact on TLR4 signaling pathways. TIFAB is shown to regulate TRAF6, a key adaptor protein in the TLR4 signaling cascade, by facilitating its degradation via lysosome‐mediated pathways. In TIFAB‐deficient cells, TRAF6 levels are significantly elevated, resulting in heightened activation of TLR4‐mediated signaling [32].

This deregulation of TLR4 signaling contributes to an inflammatory microenvironment, which disrupts the normal function of hematopoietic stem/progenitor cells (HSPCs) and promotes the progression of MDS. Furthermore, the study highlights that the activation of TLR4 signaling through stabilized TRAF6 not only exacerbates hematopoietic defects but also facilitates immune evasion and leukemic transformation. Importantly, the re‐expression of TIFAB in del(5q) MDS/AML cells suppresses TLR4 signaling, reducing inflammation and impairing leukemic cell survival. The findings underscore the central role of TLR4 signaling in the pathogenesis of del(5q) MDS and AML, demonstrating how aberrant innate immune responses driven by TIFAB deletion and TRAF6 stabilization promote disease progression. This study provides a compelling case for targeting TLR4 signaling as a potential therapeutic strategy in hematological malignancies associated with del(5q) alterations [32].

Additionally, Barreyro et al. [33] explored the interplay between dysregulated innate immune signaling and RUNX1 mutations in the progression of MDS to AML. Specifically, the study examines how the deletion of miR‐146a, a key regulator of innate immunity, enhances TLR4 signaling through TRAF6, contributing to inflammatory cascades and hematopoietic dysfunction. Using a murine model, they demonstrate that miR‐146a‐deficient hematopoietic stem and progenitor cells (HSPCs) exhibit increased susceptibility to inflammatory stress, leading to bone marrow failure and preleukemic states [33].

The introduction of a RUNX1 mutant into miR‐146a‐deficient HSPCs induces AML, highlighting a synergistic interaction between dysregulated innate immune signaling and RUNX1 mutations. Mechanistic insights reveal that miR‐146a loss amplifies TLR4‐mediated activation of NF‐kB pathways, which RUNX1 mutations exploit to drive leukemic transformation. Importantly, the study identifies that inhibiting TRAF6‐dependent signaling using a UBE2N inhibitor can suppress leukemic HSPCs, underscoring the therapeutic potential of targeting innate immune pathways in AML. This study emphasizes the critical role of TLR4 signaling in maintaining the inflammatory microenvironment that promotes leukemic progression, providing a foundation for developing targeted therapies to modulate these pathways in hematologic malignancies [33].

A recent study by Ward et al. [44] revealed that oxidized mitochondrial DNA, a DAMP frequently found in the plasma of MDS patients, can activate the inflammasome pathway mediated by the TLR9‐MyD88 interaction. This factor is released into the cytosol of the cells through NLRP3 inflammasome pyroptotic lysis, generating an inflammatory cascade boost, observed by the translocation of IRF7 and activation of interferon‐stimulated genes, which culminates in the propagation of the inflammatory trigger, including in healthy tissues, and an increased rate of cell death derived from inflammation. In addition, the interaction between mtDNA/TLR9 is related to anemia development during this inflammatory process. Thus, the interaction between ox‐mtDNA and TLR9 may signal a possible therapeutic target for MDS patients, with possible blockage of TLR9 signaling, as also shown in Table 1 [44].

In addition to developments aimed at recovering and improving the patient's inflammatory status, there are also alternative treatments that act as epigenetic modulators, for example, which act by reversing DNA hypermethylation that impairs the expression of tumor suppressor genes and contributes to oncogenesis, a factor determinant for the development and progression of MDS. Regarding treatment, there are some therapies developed for patients with MDS, such as hypomethylating agents for high‐risk patients and lenalidomide for low‐risk MDS with del 5q [24]. The use of these hypomethylating agents is crucial for the management of MDS, as they not only alleviate symptoms related to anemia and other cytopenias but also decrease the risk of transformation to AML.

These trials were crucial for thinking about the development of medicines that explore the most innate and inflammatory aspects of the disease. In fact, some studies are already being developed, such as the clinical trial carried out with patients with low‐risk MDS, in which a TLR‐2 antagonist monoclonal antibody was used, which showed that 50% of patients showed hematological improvement and 2 achieved red blood cell transfusion independence [55], demonstrated in Table 2. Reducing anemia is particularly important, as severe anemia is one of the most debilitating manifestations of MDS, leading to symptoms such as fatigue, weakness, and decreased quality of life. Furthermore, this study highlights the importance of the role of TLR‐2 in inflammation and the immune response, which suggests that inhibition of this receptor may help restore a healthier hematopoietic microenvironment.

A study using a zebrafish model of MDS with a knockout of RPS14 (uS11), also illustrated in Table 2, sought to find molecules that could improve the MDS phenotype, leading to the discovery of imiquimod, a TLR7 and TLR8 agonist, whose action alleviates anemia by stimulating the expansion of hematopoietic progenitor cells and promoting erythroid differentiation, leading RPS14 mutant MDS patients to a remarkable recovery in the anemia phenotype, due to a modulation of the inflammatory response, when compared to the control group [30]. This study with immunomodulators offers an alternative to alleviate the clinical condition of patients in relation to anemia since the cellular microenvironment could be restored by favoring the production of healthy cells.

Therefore, the broad study on immune approaches in MDS aims not only to understand how aspects of the inflammation cascade and the activation of pro‐inflammatory cytokines in immune control work, but also to carry out studies that propose the development of targeted therapies, like epi‐drugs. These strategies aim to restore the normal functioning of signaling pathways and reduce chronic inflammation, benefiting, above all, people with MDS.

### 5.1 TLRs In Acute Myeloid Leukemia—MyD88 Is Key to Understanding Its Prognosis

Acute myeloid leukemia is a thoroughly investigated disease characterized by the clonal expansion of mutated myeloid precursors in the bone marrow, interfering with the cell cycle and renewal process in the bone marrow, which over time evolves into bone marrow failure [56]. Immunity plays an important role in the course of the disease, since the presence of inflammatory stimuli, generated by mutations in genes that are important in controlling homeostasis, leads to a worse and more accelerated progression of the disease, indicating a poor prognosis. Thus, studies of new adoptive immunotherapy seek to achieve better outcomes for AML patients, but they still face barriers when it involves the overlapping of antigen expression between mutated cells and healthy cells, hindering the achievement of specific therapies [57].

In fact, as part of the immune homeostasis, the TLRs signaling, in the face of an inflammatory trigger, promotes differentiation of myeloid cells at the expense of lymphoid and red blood cells as well as the self‐renewal of hematopoietic stem cells, toward generating more white cells. This process is called common precursor synthesis deviation or “emergency myelopoiesis” [58]. However, as illustrated in Figure 2, signaling errors generated by mutations in important genes in this process, such as MyD88, have been described in hematological cancers and are known to affect the prognosis. The TLR‐MyD88 pathway in the antigen‐presenting cell is extremely important for inducing the differentiation of lymphocytes into a Th1 profile in order to generate an antitumor immune response. When overstimulated, however, this pathway stimulates proliferation of tumor cells via nuclear factor NF‐κB/JNK/ERK signaling, thereby inducing the production of cytokines, such as TNF, IL‐1α, and IL‐6, which act in a positive feedback loop to promote tumor growth [14].

In addition, this pathway mediated by the activation of MyD88 also leads to the recruitment of members of the IRAK family (IL‐1 receptor kinases), because of the interaction between IRAK‐4 and MyD88, which leads to phosphorylation and the formation of multiprotein complexes known as Myddosomes. In this process, IRAK4 activates IRAK1, which is then phosphorylated at various cellular sites and disconnects from MyD88, activating TRAF6, which participates in the activation of transforming growth factor beta‐activated kinase 1 (TAK1), which activates the NF‐κB and mitogen‐activated protein kinase (MAPK) pathways that culminate in the activation of pro‐inflammatory transcription factors, such as IL‐1, IL‐6, IL‐8, and TNF‐α, and generates a state of oxidative stress [51]. When overstimulated, this cascade of events creates an environment that not only promotes inflammation but also significantly contributes to tumor growth and progression.

Furthermore, as illustrated in Table 2, activation of toll‐like receptors through MyD88 was studied as a costimulation pathway to enhance the effector function of CAR‐T cells anti‐CD123, since the expression of this marker is shown to be high on leukemia stem cells and lower on normal hematopoietic cells. The therapy is based on an ENG T‐cell anti‐CD123 that produces bispecific antibodies consisting of two single chain variable fragments, one able to bind CD123 and the other specific for CD3ε, responsible for the activation of the T lymphocyte. This MyD88/TLR costimulation is proven to enhance the persistence and sequential killing capabilities of the ENG T cell without affecting antigen specificity [19]. In this instance, the exploration of this type of therapy exemplifies the ongoing advancements in cancer treatments, highlighting the potential for more effective and targeted therapies that improve patient outcomes while preserving the specificity of immune responses.

AML cells express leukemia‐associated antigens (LAA) that can be recognized by the immune system, in which dendritic cells play an important role, by reducing T‐cell tolerance. Curran and colleagues studied a subtype of dendritic cells, called CD8α + DCs, which could engulf the cancer cells of leukemic mice and cross‐present AML cell‐derived antigens to T cells, thereby activating them. The triggering of TLR3, preferentially expressed on CD8α + dendritic cells, by the adaptor molecule TRIFF, activates the transcription factors of interferon regulatory factor 3 and NF‐κB, resulting in the production of INF‐β, leading to an increased immune response [40], indicated in Table 1. By modulating immune responses, it highlighted the critical balance between effective antigen presentation and the potential for immune evasion in the tumor microenvironment.

The use of TLR agonists has been widely explored by researchers due to their strong potential as a therapeutic tool. Starring Taghiloo et al. [28] a study in AML murine models with the TLR7/8 agonist BEZ235 in combination with R848 showed an increased frequency of antitumor immune cells and a significantly improved survival analysis [28], shown in Table 2. His in vivo research demonstrated that only the R848 agonist had the capacity to reprogram AML cells into a phenotype conducive to IFN production, thereby aiding in tumor defense. In this study, exhibited in Table 2, the authors advocate for the activation of plasmacytoid dendritic cells (pCDs), known to be elevated in AML patients and apoptosis inducers, through R848 treatment. They observed an augmentation in IFN beta production, accompanied by an increase in CD38 antibody levels, resulting in programmed cell death of AML cells. Another study, in line with the aforementioned findings, conducted by Ronsley et al. [59], illustrates that the intravenous administration of the TLR9 agonist GNKG168 to pediatric ALL patients in remission triggers immunological changes, leading to a notable enhancement in immune responses characterized by increased T and B cell production, improving the ability of tumor recognition by immune cells [29, 59], as indicated in Table 2. Both studies have demonstrated that TLR agonists enhance antitumor responses by promoting differentiation and growth inhibition of leukemic cells, activating immune responses through dendritic cell maturation, and increasing the production of pro‐inflammatory cytokines, ultimately improving patient outcomes and survival rates.

Aref et al. [45], studying the clinical implications of TLR2 Arg753Gln, TLR4 Asp299Gly, and TLR4 Thr399Ile polymorphisms in AML patients, reported that TLR2 (Arg753Gln) GG polymorphisms are significantly associated with shorter OS and disease‐free survival. Additionally, TLR4 polymorphisms primarily impact DFS but not OS. These associations may be attributed to the role of TLR2 and TLR4 in promoting inflammation and immune evasion. TLR2, through MyD88‐mediated signaling, can activate the NF‐κB pathway, resulting in increased pro‐inflammatory cytokine production, tumor cell proliferation, and survival.

Similarly, TLR4 signaling via its adaptor molecule TRIF contributes to the production of type I interferons and inflammatory cytokines, fostering an immunosuppressive tumor microenvironment. These mechanisms highlight the dual role of these receptors in modulating immune responses and influencing AML progression and prognosis. These results suggest that TLR2 and TLR4 could serve as prognostic biomarkers and potential therapeutic targets in AML, as their elevated expressions are associated with adverse clinical outcomes and more aggressive disease characteristics [45], data presented in Table 1. This way, it is shown how these biomarkers can be utilized in clinical practice to stratify patient risk at diagnosis, guiding treatment decisions and monitoring responses to therapy; specifically, elevated levels of TLR2 and TLR4 may indicate a more aggressive disease phenotype and poorer prognosis, allowing clinicians to tailor more aggressive treatment strategies or consider novel therapeutic approaches targeting these pathways to improve patient outcomes.

Focusing on the development of an AML treatment, Wooil et al. [26] investigated the potential of bioengineered yeast vacuoles expressing TLR2‐binding peptides (VacT2BP) as a drug carrier for daunorubicin. The findings demonstrated that VacT2BP induced a pro‐inflammatory response through TLR2 activation, leading to enhanced immunogenicity and antitumor efficacy. Furthermore, VacT2BP effectively encapsulated and delivered daunorubicin to AML cells, resulting in potent cytotoxicity and apoptosis induction. Notably, the delivery of daunorubicin via VacT2BP exhibited superior therapeutic outcomes with reduced toxicity compared to free daunorubicin, leading to enhanced tumor regression and prolonged survival in AML mouse models. These findings suggest that VacT2BP holds promise as a drug delivery system for AML treatment, leveraging TLR2‐mediated immune stimulation and efficient drug transport to enhance therapeutic efficacy and overcome drug resistance [26], shown in Table 2.

In the same field, Schmitt et al. [42] found that combining bacterial flagellin, a TLR5 ligand, with an αCD40 antibody construct and specific antigens significantly enhances dendritic cell (DC) maturation and T‐cell activation. The fusion construct increased DC maturation markers (CD80, CD86, and MHC class II) and cytokine production (IL‐12 and TNF‐alpha) compared to controls. T cells co‐cultured with DCs treated with the fusion construct exhibited higher proliferation and IFN‐γ production. Furthermore, as described in Table 2, the construct targeting leukemia‐specific antigens led to a significant increase in leukemia‐specific T‐cell activation compared to viral antigens. These results underscore the potential of targeting TLR5 to enhance DC maturation and antigen presentation, offering a promising strategy for personalized immunotherapy against leukemia [42].

In another instance, based on the premise that stem cell transplantation underscores the immune‐responsive nature of AML, Sehmus et al. [38] investigated the impact of palmitoylated proteins carried by extracellular vesicles (EVs) from AML in driving the differentiation of myeloid‐derived suppressor cells (MDSCs) via the TLR2/Akt/mTOR signaling pathway. They found that palmitoylated proteins on AML‐derived EVs activate TLR2 signaling, leading to downstream Akt/mTOR pathway activation, crucial for MDSC proliferation and immunosuppressive function in the AML microenvironment. Inhibiting TLR2 or Akt/mTOR signaling mitigated MDSC differentiation, suggesting a potential therapeutic strategy to counter MDSC‐mediated immune suppression in AML, highlighting the significance of EV‐associated palmitoylated proteins in disrupting leukemogenesis and improving immune responses, thereby enhancing antitumor immune responses and improving patient outcomes in acute myeloid leukemia [38], as illustrated in Table 1.

Focusing on the genetics of population, a study by Pawlowska et al. [60] shows that polymorphisms in genes encoding TLRs were linked to clinical parameters in European AML patients. This study showed that AML patients with favorable or intermediate risk per the European LeukemiaNet (ELN) classification were more likely to carry the rs187084 C allele in the TLR9 gene, which was less frequent in those with unfavorable prognosis, suggesting a protective role against high‐risk AML. Additionally, infectious complications during AML therapy were more frequently observed in patients with the rs5743305 AA genotype in the TLR3 gene. Since this receptor recognizes viral dsRNA, these patients may experience receptor malfunction, increasing their susceptibility to viral infections. Moreover, extramedullary metastases were more common in patients with the rs3775296 T allele in the TLR3 gene and the rs4986790 G allele in the TLR4 gene, whereas the rs3775291 A allele in TLR3 and the rs187084 C allele in TLR9 were less frequent in these patients [60]. In this way, identifying specific genetic variants associated with prognosis and susceptibility to infections and immune modulations can guide personalized treatment strategies, improve risk stratification, and enhance patient monitoring, ultimately leading to better clinical outcomes.

It is very important to highlight the role of TLRs in the proliferation and differentiation of hematopoietic stem cells, in which their transitory activation leads to the expression of numerous genes related to the immune response and the identification of self‐antigens, to control autoimmune events. However, chronic stimulation of TLRs leads to exacerbation of the immune response, leading to cell exhaustion, bone marrow failure, and aggravation of the disease [51]. Understanding the fine‐tuning that TLRs exert on the immune mechanism and the range of variations that can occur in this regard is crucial to the development of new approaches for the treatment of AML.

## 6 TLRs In Chronic Myeloid Leukemia—How Does Chronic Inflammatory Stimuli Promote the Progression?

Chronic myeloid leukemia (CML) is mainly characterized by the formation of the BCR‐ABL1 oncogene from a translocation between the long arms of chromosomes 9 and 22. It is estimated that CML affects elderly patients most often, but it can occur in all age groups and is the cause of approximately 15% of new leukemia diagnoses in adults. CML is subdivided into 3 phases, the initial one being called chronic, which if left untreated can progress to an accelerated phase and reach a blast phase, with unfavorable prognostic factors (increased blast count, cytogenetic alterations, splenomegaly) [61]. About DNA methylation, the transitions that occur in blastic crisis (whether myeloid or lymphoid) are indicated as episodes of hypermethylation, which are related to the predominance of changes in gene expression at this stage of the disease. In addition, there are alterations in DNA methylation in the chronic phase, which is maintained in the transformation to the blastic phase as hypomethylation [7].

In CML, the high expression of pro‐inflammatory mediators such as TNF, interferon (IFN), IL1‐R has been indicated, as well as the signaling of Toll‐like receptors (TLR) in relation to the actions of TP53 and the processes of cell cycle and apoptosis. Thus, in a study carried out with a culture of K562 CML cells, it was shown that stimulation by lipopolysaccharide (LPS) mainly increased the expression of sirtuin 1 (SIRT 1), which led to a decrease in the expression of TLR4 based on a reduction in the production of pro‐inflammatory cytokines. This reduction in TLR4 ended up limiting the action of the nuclear factor κB (NF‐κB) signaling axis and the generation of reactive oxygen species [21, 62], shown in Table 1 and Table 2. Therefore, understanding these mechanisms highlights how dysregulation of TLR signaling can contribute to CML progression and suggests potential therapeutic targets for intervention to inhibit tumor growth.

Considering that the leukemic process of CML is enhanced by events such as increased cell survival and genomic instability, strategies such as CRISPR technology have been evaluated. In fact, Vuelta et al. [23] applied the CRISPR‐Trap strategy, which integrates the CRISPR/Cas9 system with a non‐viral gene capture donor inserted via homologous recombination. This assay annulled the expression of the oncogene by inserting a fluorescent reporter gene into the BCR/ABL, as displayed in Figure 2 and Table 2, coding sequence to select the edited hematopoietic cells of CML. Of note, there was a decrease of over 80% in BCR/ABL expression levels [23]. The application of CRISPR technology offers a transformative approach to CML treatment by effectively targeting and eliminating the BCR/ABL oncogene, which is crucial for leukemic cell survival. Not only does it significantly reduce oncogene expression, but it also holds the potential to restore normal hematopoiesis, can improve patient prognosis, and provide a promising avenue for addressing resistance to conventional therapies.

Sébastien et al. [22] carried out an analysis of 60 patients with CML who discontinued treatment with tyrosine kinase inhibitors (TKI), 30 of whom were going through a period of remission without treatment and the remaining 30 had molecular recurrence, as well as 10 control patients. As illustrated in Table 2, patients in the recurrence group showed increased expression of TLR1, TLR6, and TLR8 receptors, so that NOD‐like and TNF‐α signaling were increased, and they are commonly associated with promoting a protective CML microenvironment [22]. This study suggests that the presence of a protective microenvironment, for example, may hinder the effectiveness of conventional treatments such as TKIs. Thus, therapeutic strategies aimed at modifying this microenvironment or integrating immunotherapy may be necessary to improve outcomes in relapsed patients.

In conclusion, TLRs play a significant role in the pathophysiology of CML. The high expression of pro‐inflammatory mediators and TLR signaling is associated with critical processes such as cell cycle regulation and apoptosis, whose modulation can limit inflammatory signaling pathways and decrease ROS production. Additionally, increased expression of these markers in patients with molecular recurrence highlights the role of TLRs in promoting a protective CML microenvironment, underscoring the potential of targeting TLR pathways as a therapeutic strategy in CML.

## 7 Conclusion

In conclusion, the complex interaction between TLRs and hematological malignancies highlights the fundamental role of innate immunity in the development of both disease progression and prognosis. TLRs act as critical sensors, responding to microbial patterns and damage‐associated molecules, thereby initiating inflammatory cascades. This dynamic role is evidenced in hematological cancers such as MDS, AML, and CML, underscoring the complexity of their function. Each TLR plays a critical role in modulating specific aspects of the immune system. In each disease, these receptors are crucial as prognostic factors, indicating varying impacts on overall survival, disease‐free survival, and the development of targeted therapies such as TLR agonists.

For instance, dysregulation of TLR signaling pathways contributes to disease progression and poor prognosis in MDS and AML. Therapeutic strategies that modulate TLR activity, including TLR agonists and antagonists, have shown promise in preclinical and clinical studies, offering new avenues for treatment. This way, the exploration of TLR agonists and antagonists as therapeutic modalities demonstrates the potential for drawing on immune responses against cancer cells, showing as a promising field of experimentation where more attention is expected, mainly due to the use of purine nucleoside phosphorylase inhibitors with immunotherapies that can enhance antitumor response.

Notably, the MyD88 pathway emerged as a critical component, influencing prognosis in acute myeloid leukemia and guiding innovative immunotherapies. Interestingly, variations in pathways regulating the expression of various TLRs, as discussed throughout this article, can significantly impact individual susceptibility to infections and shape the clinical trajectory of hematological malignancies. This genetic aspect provides a basis for personalized medicine approaches, where therapies have been and will continue to be tailored based on a patient's TLR profile.

The study of TLR expression and signaling as markers must be investigated in the condition of each disease, as its role as a prognostic marker is not entirely clear, but its deregulation can confer a proliferative advantage to cancer cells. This way, the comprehensive understanding of TLR modulation provides a basis for the development of targeted therapies, emphasizing the intricate balance between immune response and disease progression. These insights hold significant promise for advancing hematologic cancer treatments and improving patient outcomes.

## Conflicts of Interest

The authors declare no conflicts of interest.

## Data Availability

Data sharing not applicable to this article as no datasets were generated or analyzed during the current study.

## References

[1] J. Zindel and P. Kubes , “DAMPs, PAMPs, and LAMPs in Immunity and Sterile Inflammation,” Annual Review of Pathology 15 (2020): 493–518. 10.1146/annurev-pathmechdis-012419-032847 31675482

[2] P. Behzadi , H. A. García‐Perdomo , and T. M. Karpiński , “Toll‐Like Receptors: General Molecular and Structural Biology,” Journal of Immunology Research 2021 (2021): 9914854, 10.1155/2021/9914854. 34195298 PMC8181103

[3] S. Akira , S. Uematsu , and O. Takeuchi , “Pathogen Recognition and Innate Immunity,” Cell 124, no. 4 (2006): 783–801. 16497588 10.1016/j.cell.2006.02.015

[4] O. Takeuchi and S. Akira , “Pattern Recognition Receptors and Inflammation,” Cell 140, no. 6 (2010): 805–820. 20303872 10.1016/j.cell.2010.01.022

[5] K. R. Balka and D. De Nardo , “Understanding Early TLR Signaling Through the Myddosome,” Journal of Leukocyte Biology 105, no. 2 (2019): 339–351. 30256449 10.1002/JLB.MR0318-096R

[6] E. M. Y. Moresco , D. LaVine , and B. Beutler , “Toll‐Like Receptors,” Current Biology 21, no. 13 (2011): R488–R493. 21741580 10.1016/j.cub.2011.05.039

[7] T. K. Ko , A. Javed , K. L. Lee , et al., “An Integrative Model of Pathway Convergence in Genetically Heterogeneous Blast Crisis Chronic Myeloid Leukemia,” Blood 135, no. 26 (2020): 2337–2353. 32157296 10.1182/blood.2020004834

[8] P. Behzadi , D. Chandran , C. Chakraborty , et al., “The Dual Role of Toll‐Like Receptors in COVID‐19: Balancing Protective Immunity and Immunopathogenesis,” International Journal of Biological Macromolecules 284, no. Pt 2 (2025): 137836, 10.1016/j.ijbiomac.2024.137836. 39613064

[9] T. Kawai and S. Akira , “The Role of Pattern‐Recognition Receptors in Innate Immunity: Update on Toll‐Like Receptors,” Nature Immunology 11, no. 5 (2010): 373–384, https://www.nature.com/articles/ni.1863. 20404851 10.1038/ni.1863

[10] D. A. Monlish , S. T. Bhatt , and L. G. Schuettpelz , “The Role of Toll‐Like Receptors in Hematopoietic Malignancies,” Frontiers in Immunology 7 (2016): 223495. 10.3389/fimmu.2016.00390 PMC5039188 27733853

[11] Y. Mokhtari , A. Pourbagheri‐Sigaroodi , P. Zafari , N. Bagheri , S. H. Ghaffari , and D. Bashash , “Toll‐Like Receptors (TLRs): An Old Family of Immune Receptors With a New Face in Cancer Pathogenesis,” Journal of Cellular and Molecular Medicine 25, no. 2 (2021): 639–651, https://onlinelibrary.wiley.com/doi/full/10.1111/jcmm.16214. 33336901 10.1111/jcmm.16214 PMC7812258

[12] S. Mukherjee and J. Bayry , “The Yin and Yang of TLR4 in COVID‐19,” Cytokine & Growth Factor Reviews 82 (2025): 70–85, 10.1016/j.cytogfr.2024.10.001. 39490235

[13] S. Bagchi , R. Yuan , and E. G. Engleman , “Immune Checkpoint Inhibitors for the Treatment of Cancer: Clinical Impact and Mechanisms of Response and Resistance,” Annual Review of Pathology: Mechanisms of Disease 16 (2021): 223–249. 10.1146/annurev-pathol-042020-042741 33197221

[14] M. Y. Park , S. E. Ha , H. H. Kim , et al., “Scutellarein Inhibits LPS‐Induced Inflammation Through NF‐κB/MAPKs Signaling Pathway in RAW264.7 Cells,” Molecules 27, no. 12 (2022): 3782. 35744907 10.3390/molecules27123782 PMC9227861

[15] S. Mukherjee , S. Huda , and S. P. Sinha Babu , “Toll‐Like Receptor Polymorphism in Host Immune Response to Infectious Diseases: A Review,” Scandinavian Journal of Immunology 90, no. 1 (2019): e12771, 10.1111/sji.12771. 31054156

[16] I. Aladzsity , M. Kovács , Á. Semsei , et al., “Comparative Analysis of IL6 Promoter and Receptor Polymorphisms in Myelodysplasia and Multiple Myeloma,” Leukemia Research 33, no. 11 (2009): 1570–1573. 19406470 10.1016/j.leukres.2009.03.009

[17] S. Mukherjee , R. Patra , P. Behzadi , A. Masotti , A. Paolini , and M. Sarshar , “Toll‐Like Receptor‐Guided Therapeutic Intervention of Human Cancers: Molecular and Immunological Perspectives,” Frontiers in Immunology 14 (2023): 1244345, 10.3389/fimmu.2023.1244345. 37822929 PMC10562563

[18] Z. Urban‐Wojciuk , M. M. Khan , B. L. Oyler , et al., “The Role of Tlrs in Anti‐Cancer Immunity and Tumor Rejection,” Frontiers in Immunology 10 (2019): 484631. 10.3389/fimmu.2019.02388 PMC6817561 31695691

[19] A. Vaidya , E. Doherty , X. Wu , et al., “Improving the Anti‐Acute Myeloid Leukemia Activity of CD123‐Specific Engager T Cells by MyD88 and CD40 Costimulation,” Haematologica 108, no. 4 (2023): 1039–1052, https://haematologica.org/article/view/haematol.2021.279301. 35899386 10.3324/haematol.2021.279301 PMC10071120

[20] S. Baakhlagh , B. Kashani , Z. Zandi , et al., “Toll‐Like Receptor 4 Signaling Pathway Is Correlated With Pathophysiological Characteristics of AML Patients and Its Inhibition Using TAK‐242 Suppresses AML Cell Proliferation,” International Immunopharmacology 90 (2021): 107202. 33278749 10.1016/j.intimp.2020.107202

[21] L. Wang , M. Wang , H. Dou , W. Lin , and L. Zou , “Sirtuin 1 Inhibits Lipopolysaccharide‐Induced Inflammation in Chronic Myelogenous Leukemia k562 Cells Through Interacting With the Toll‐Like Receptor 4‐Nuclear Factor κ B‐Reactive Oxygen Species Signaling Axis,” Cancer Cell International 20, no. 1 (2020): 1–10, 10.1186/s12935-020-1152-z. 32165863 PMC7059700

[22] R. Sébastien , C. Sticht , M. Pfirrmann , et al., “The EUROSKI Biomarker Study: Analyzing the Mechanisms of Treatment‐Free Remission in Chronic Myeloid Leukemia,” Annals of Oncology 28 (2017): v367.

[23] E. Vuelta , J. L. Ordoñez , D. J. Sanz , et al., “CRISPR/Cas9‐Directed Gene Trap Constitutes a Selection System for Corrected BCR/ABL Leukemic Cells in CML,” International Journal of Molecular Sciences 23, no. 12 (2022): 6386. 35742831 10.3390/ijms23126386 PMC9224210

[24] E. Jabbour , N. J. Short , G. Montalban‐Bravo , et al., “Randomized Phase 2 Study of Low‐Dose Decitabine vs. Low‐Dose Azacitidine in Lower‐Risk MDS and MDS/MPN,” Blood 130, no. 13 (2017): 1514–1522, 10.1182/blood-2017-06-788497. 28774880 PMC5620419

[25] S. Li , J. C. Yao , K. A. Oetjen , et al., “IL‐1β Expression in Bone Marrow Dendritic Cells Is Induced by TLR2 Agonists and Regulates HSC Function,” Blood 140, no. 14 (2022): 1607–1620. 35675516 10.1182/blood.2022016084 PMC9707400

[26] W. Choi , W. R. Shin , Y. H. Kim , and J. Min , “Inducing a Proinflammatory Response With Bioengineered Yeast Vacuoles With TLR2‐Binding Peptides (VacT2BP) as a Drug Carrier for Daunorubicin Delivery,” ACS Applied Materials & Interfaces 15, no. 35 (2023): 41258–41270. 37615983 10.1021/acsami.3c06669

[27] D. A. Monlish , Z. J. Greenberg , S. T. Bhatt , et al., “TLR2/6 Signaling Promotes the Expansion of Premalignant Hematopoietic Stem and Progenitor Cells in the NUP98‐HOXD13 Mouse Model of MDS,” Experimental Hematology 88 (2020): 42–55. 32652111 10.1016/j.exphem.2020.07.001 PMC7673652

[28] S. Taghiloo , A. Ajami , R. Alizadeh‐Navaei , and H. Asgarian‐Omran , “Combination Therapy of Acute Myeloid Leukemia by Dual PI3K/mTOR Inhibitor BEZ235 and TLR‐7/8 Agonist R848 in Murine Model,” International Immunopharmacology 125, no. Pt B (2023): 111211. 37956488 10.1016/j.intimp.2023.111211

[29] K. Fatehchand , P. Mehta , C. B. Colvin , et al., “Activation of Plasmacytoid Dendritic Cells Promotes AML‐Cell Fratricide,” Oncotarget 12, no. 9 (2021): 878–890. 33953842 10.18632/oncotarget.27949 PMC8092344

[30] O. A. Peña , A. Lubin , C. Hockings , et al., “TLR7 Ligation Augments Hematopoiesis in Rps14 (uS11) Deficiency via Paradoxical Suppression of Inflammatory Signaling,” Blood Advances 5, no. 20 (2021): 4112–4124, 10.1182/bloodadvances.2020003055. 34432872 PMC8945628

[31] A. Y. F. Kam , S. O. Piryani , C. M. McCall , H. S. Park , D. A. Rizzieri , and P. L. Doan , “Targeting High Mobility Group Box‐1 (HMGB1) Promotes Cell Death in Myelodysplastic Syndrome,” Clinical Cancer Research 25, no. 13 (2019): 4055–4167. 10.1158/1078-0432.CCR-18-3517 PMC6800136 30952643

[32] M. E. Varney , M. Niederkorn , H. Konno , et al., “Loss of Tifab, a del(5q) MDS Gene, Alters Hematopoiesis Through Derepression of Toll‐Like Receptor‐TRAF6 Signaling,” Journal of Experimental Medicine 212, no. 11 (2015): 1967–1985. 26458771 10.1084/jem.20141898 PMC4612089

[33] L. Barreyro , A. M. Sampson , K. Hueneman , et al., “Dysregulated Innate Immune Signaling Cooperates With RUNX1 Mutations to Transform an MDS‐Like Disease to AML,” IScience 27, no. 6 (2024): 109809. 38784013 10.1016/j.isci.2024.109809 PMC11112336

[34] S. O. Abegunde , R. Buckstein , R. A. Wells , and M. J. Rauh , “An Inflammatory Environment Containing TNFα Favors Tet2 ‐Mutant Clonal Hematopoiesis,” Experimental Hematology 59 (2018): 60–65, 10.1016/j.exphem.2017.11.002. 29195897

[35] S. Padma , R. Patra , P. S. Sen Gupta , S. K. Panda , M. K. Rana , and S. Mukherjee , “Cell Surface Fibroblast Activation Protein‐2 (Fap2) of *Fusobacterium nucleatum* as a Vaccine Candidate for Therapeutic Intervention of Human Colorectal Cancer: An Immunoinformatics Approach,” Vaccine 11, no. 3 (2023): 525, 10.3390/vaccines11030525. PMC10056511 36992108

[36] C. Rizzello , V. Cancila , S. Sangaletti , et al., “Intracellular Osteopontin Protects From Autoimmunity‐Driven Lymphoma Development Inhibiting TLR9‐MYD88‐STAT3 Signaling,” Molecular Cancer 21, no. 1 (2022): 215. 36503430 10.1186/s12943-022-01687-6 PMC9743519

[37] X. Chen , Q. Zhang , Y. Luo , et al., “High‐Dose Irradiation in Combination With Toll‐Like Receptor 9 Agonist CpG Oligodeoxynucleotide 7909 Downregulates PD‐L1 Expression via the NF‐κB Signaling Pathway in Non‐Small Cell Lung Cancer Cells,” Oncotargets and Therapy 9 (2016): 6511–6518. 27799798 10.2147/OTT.S116629 PMC5085295

[38] S. Tohumeken , R. Baur , M. Bottcher , et al., “Palmitoylated Proteins on AML‐Derived Extracellular Vesicles Promote Myeloid‐Derived Suppressor Cell Differentiation via TLR2/Akt/mTOR Signaling,” Cancer Research 80, no. 17 (2020): 3663–3676. 32605996 10.1158/0008-5472.CAN-20-0024

[39] R. T. G. de Oliveira , J. V. A. Cordeiro , B. F. Vitoriano , et al., “ERVs‐TLR3‐IRF Axis is Linked to Myelodysplastic Syndrome Pathogenesis,” Medical Oncology 38, no. 3 (2021): 27. 33594613 10.1007/s12032-021-01466-1

[40] E. Curran , L. Corrales , and J. Kline , “Targeting the Innate Immune System as Immunotherapy for Acute Myeloid Leukemia,” Frontiers in Oncology 5 (2015): 125778. 10.3389/fonc.2015.00083 PMC4391043 25914882

[41] L. Shi , Y. Zhao , C. Fei , et al., “Cellular Senescence Induced by S100A9 in Mesenchymal Stromal Cells Through NLRP3 Inflammasome Activation,” Aging 11, no. 21 (2019): 9626–9642, https://www.aging‐us.com/article/102409. 31727865 10.18632/aging.102409 PMC6874461

[42] S. Schmitt , S. Tahk , A. Lohner , et al., “Fusion of Bacterial Flagellin to a Dendritic Cell‐Targeting αCD40 Antibody Construct Coupled With Viral or Leukemia‐Specific Antigens Enhances Dendritic Cell Maturation and Activates Peptide‐Responsive T Cells,” Frontiers in Immunology 11 (2020): 602802. 33281829 10.3389/fimmu.2020.602802 PMC7689061

[43] J. Qian , H. Meng , B. Lv , et al., “High Expression Levels of TLR9 and PD‐L1 Indicates a Poor Prognosis in Patients With Angioimmunoblastic T‐Cell Lymphoma: A Retrospective Study of 88 Cases in a Single Center,” Journal of Cancer 11, no. 1 (2020): 57–68. 31892973 10.7150/jca.37033 PMC6930404

[44] G. A. Ward , R. P. Dalton , B. S. Meyer , et al., “Oxidized Mitochondrial DNA Engages TLR9 to Activate the NLRP3 Inflammasome in Myelodysplastic Syndromes,” International Journal of Molecular Sciences 24, no. 4 (2023): 3896, https://www.mdpi.com/1422–0067/24/4/3896/htm. 36835307 10.3390/ijms24043896 PMC9966808

[45] S. Aref , A. S. Abd Elmaksoud , S. A. Elaziz , M. Mabed , and M. Ayed , “Clinical Implication of Toll‐Like Receptors (TLR2 and TLR4) in Acute Myeloid Leukemia Patients,” Asian Pacific Journal of Cancer Prevention 21, no. 11 (2020): 3177–3183. 33247673 10.31557/APJCP.2020.21.11.3177 PMC8033142

[46] P. Khalilian , N. Eskandari , M. J. Sharifi , M. Soltani , and P. Nematollahi , “Toll‐Like Receptor 4, 2, and Interleukin 1 Receptor Associated Kinase4: Possible Diagnostic Biomarkers in Myelodysplastic Syndrome Patients,” Advanced Biomedical Research 13, no. 1 (2024): 17, https://journals.lww.com/adbm/fulltext/2024/02260/toll_like_receptor_4,_2,_and_interleukin_1.4.aspx. 38525404 10.4103/abr.abr_67_23 PMC10958736

[47] A. Di Lorenzo , E. Bolli , L. Tarone , F. Cavallo , and L. Conti , “Toll‐Like Receptor 2 at the Crossroad Between Cancer Cells, the Immune System, and the Microbiota,” International Journal of Molecular Sciences 21 (2020): 9418. 33321934 10.3390/ijms21249418 PMC7763461

[48] R. Alaggio , C. Amador , I. Anagnostopoulos , et al., “The 5th Edition of the World Health Organization Classification of Haematolymphoid Tumours: Lymphoid Neoplasms,” Leukemia 36, no. 7 (2022): 1720–1748. 35732829 10.1038/s41375-022-01620-2 PMC9214472

[49] L. Barreyro , T. M. Chlon , and D. T. Starczynowski , “Chronic Immune Response Dysregulation in MDS Pathogenesis,” Blood 132, no. 15 (2018): 1553–1560, 10.1182/blood-2018-03-784116. 30104218 PMC6182269

[50] H. Li , F. Hu , R. P. Gale , M. A. Sekeres , and Y. Liang , “Myelodysplastic Syndromes,” Nature Reviews. Disease Primers 8, no. 1 (2022): 74. 10.1038/s41572-022-00402-5 36396662

[51] T. Muto , C. S. Walker , K. Choi , et al., “Adaptive Response to Inflammation Contributes to Sustained Myelopoiesis and Confers a Competitive Advantage in Myelodysplastic Syndrome HSCs,” Nature Immunology 21, no. 5 (2020): 535–545. 32313245 10.1038/s41590-020-0663-z PMC7402480

[52] L. C. Paracatu , D. A. Monlish , Z. J. Greenberg , et al., “Toll‐Like Receptor and Cytokine Expression Throughout the Bone Marrow Differs Between Patients With Low‐ and High‐Risk Myelodysplastic Syndromes,” Experimental Hematology 110 (2022): 47–59. 35367529 10.1016/j.exphem.2022.03.011 PMC9590644

[53] A. Gil‐Perez and G. Montalban‐Bravo , “Management of Myelodysplastic Syndromes After Failure of Response to Hypomethylating Agents,” Therapeutic Advances in Hematology 10 (2019): 1–18. 10.1177/2040620719847059 PMC6515843 31156799

[54] C. Giallongo , I. Dulcamare , S. Giallongo , et al., “MacroH2A1.1 as a Crossroad Between Epigenetics, Inflammation and Metabolism of Mesenchymal Stromal Cells in Myelodysplastic Syndromes,” Cell Death & Disease 14 (2023): 10, https://www.nature.com/articles/s41419‐023‐06197‐x. 37852977 10.1038/s41419-023-06197-x PMC10584900

[55] D. A. Sallman and A. List , “The Central Role of Inflammatory Signaling in the Pathogenesis of Myelodysplastic Syndromes,” Blood 133, no. 10 (2019): 1039–1048. 30670444 10.1182/blood-2018-10-844654 PMC7022316

[56] M. R. Shaikh , G. Haider , P. Memon , et al., “Cytogenetic Abnormalities in Acute Myeloid Leukaemia Patients,” Journal of Ayub Medical College, Abbottabad 32, no. 1 (2020): 33–37. 32468751

[57] H. Ma , S. P. Iyer , S. Parmar , and Y. Gong , “Adoptive Cell Therapy for Acute Myeloid Leukemia,” Leukemia & Lymphoma 60, no. 6 (2019): 1370–1380. 30628504 10.1080/10428194.2018.1553300

[58] L. Strauss , V. Guarneri , A. Gennari , and A. Sica , “Implications of Metabolism‐Driven Myeloid Dysfunctions in Cancer Therapy,” Cellular & Molecular Immunology 18, no. 4 (2020): 829–841, https://www.nature.com/articles/s41423‐020‐00556‐w. 33077904 10.1038/s41423-020-00556-w PMC7570408

[59] R. Ronsley , A. Kariminia , B. Ng , et al., “The TLR9 Agonist (GNKG168) Induces a Unique Immune Activation Pattern In Vivo in Children With Minimal Residual Disease Positive Acute Leukemia: Results of the TACL T2009‐008 Phase I Study,” Pediatric Hematology and Oncology 36, no. 8 (2019): 468–481. 31530240 10.1080/08880018.2019.1667461

[60] K. Wicherska‐Pawłowska , K. Bogunia‐Kubik , B. Kuszczak , et al., “Polymorphisms in the Genes Coding for TLRs, NLRs and RLRs Are Associated With Clinical Parameters of Patients With Acute Myeloid Leukemia,” International Journal of Molecular Sciences 23 (2022): 9593. 36076988 10.3390/ijms23179593 PMC9455872

[61] P. A. Brown , B. Shah , A. Advani , et al., “Acute Lymphoblastic Leukemia, Version 2.2021, NCCN Clinical Practice Guidelines in Oncology,” Journal of the National Comprehensive Cancer Network 19, no. 9 (2021): 1079–1109. 34551384 10.6004/jnccn.2021.0042

[62] V. Schäfer , H. E. White , G. Gerrard , et al., “Assessment of Individual Molecular Response in Chronic Myeloid Leukemia Patients With Atypical BCR‐ABL1 Fusion Transcripts: Recommendations by the EUTOS Cooperative Network,” Journal of Cancer Research and Clinical Oncology 147, no. 10 (2021): 3081–3089, 10.1007/s00432-021-03569-8. 33677711 PMC8397658

